# Niemann-Pick C Disease Gene Mutations and Age-Related Neurodegenerative Disorders

**DOI:** 10.1371/journal.pone.0082879

**Published:** 2013-12-30

**Authors:** Michael Zech, Georg Nübling, Florian Castrop, Angela Jochim, Eva C. Schulte, Brit Mollenhauer, Peter Lichtner, Annette Peters, Christian Gieger, Thorsten Marquardt, Marie T. Vanier, Philippe Latour, Hans Klünemann, Claudia Trenkwalder, Janine Diehl-Schmid, Robert Perneczky, Thomas Meitinger, Konrad Oexle, Bernhard Haslinger, Stefan Lorenzl, Juliane Winkelmann

**Affiliations:** 1 Neurologische Klinik und Poliklinik, Klinikum rechts der Isar, Technische Universität München, Munich, Germany; 2 Institut für Humangenetik, Helmholtz Zentrum München, Munich, Germany; 3 Institute of Epidemiology II, Helmholtz Zentrum München, Munich, Germany; 4 Institute of Genetic Epidemiology, Helmholtz Zentrum München, Munich, Germany; 5 Institut für Humangenetik, Technische Universität München, Munich, Germany; 6 Neurologische Klinik und Poliklinik, Klinikum der Universität München, Ludwig-Maximilians-Universität München, Munich, Germany; 7 Paracelsus-Elena-Klinik, Kassel, Germany; 8 Department of Psychiatry and Psychotherapy, Technische Universität München, Munich, Germany; 9 Neuroepidemiology and Ageing Research Unit, School of Public Health, Faculty of Medicine, The Imperial College of Science, Technology and Medicine, London, United Kingdom; 10 West London Cognitive Disorders Treatment and Research Unit, West London Mental Health Trust, London, United Kingdom; 11 Klinik für Psychiatrie und Psychotherapie, Universität Regensburg, Regensburg, Germany; 12 Neurochirurgische Klinik, Georg-August-Universität, Göttingen, Germany; 13 Klinik für Kinder- und Jugendmedizin, Universitätsklinikum Münster, Münster, Germany; 14 Laboratoire Gillet-Mérieux, CBPE, Hospices Civils de Lyon, Lyon, France; 15 UF de Neurogénétique Moléculaire, CBPE, Hospices Civils de Lyon, Lyon, France; 16 Munich Cluster for Systems Neurology, SyNergy, Munich, Germany; 17 Department of Neurology and Neurological Sciences and Center for Sleep Sciences and Medicine, Stanford University School of Medicine, Palo Alto, California, United States of America; Centre Hospitalier Universitaire Vaudois (CHUV), Switzerland

## Abstract

Niemann-Pick type C (NPC) disease is a rare autosomal-recessively inherited lysosomal storage disorder caused by mutations in *NPC1* (95%) or *NPC2*. Given the highly variable phenotype, diagnosis is challenging and particularly late-onset forms with predominantly neuropsychiatric presentations are likely underdiagnosed. Pathophysiologically, genetic alterations compromising the endosomal/lysosomal system are linked with age-related neurodegenerative disorders. We sought to examine a possible association of rare sequence variants in *NPC1* and *NPC2* with Parkinson's disease (PD), frontotemporal lobar degeneration (FTLD) and progressive supranuclear palsy (PSP), and to genetically determine the proportion of potentially misdiagnosed NPC patients in these neurodegenerative conditions. By means of high-resolution melting, we screened the coding regions of *NPC1* and *NPC2* for rare genetic variation in a homogenous German sample of patients clinically diagnosed with PD (n = 563), FTLD (n = 133) and PSP (n = 94), and 846 population-based controls. The frequencies of rare sequence variants in *NPC1*/*2* did not differ significantly between patients and controls. Disease-associated *NPC1*/*2* mutations were found in six PD patients (1.1%) and seven control subjects (0.8%), but not in FTLD or PSP. All rare variation was detected in the heterozygous state and no compound heterozygotes were observed. Our data do not support the hypothesis that rare *NPC1*/*2* variants confer susceptibility for PD, FTLD, or PSP in the German population. Misdiagnosed NPC patients were not present in our samples. However, further assessment of NPC disease genes in age-related neurodegeneration is warranted.

## Introduction

Niemann-Pick type C (NPC) disease (OMIM*257220 and OMIM*607625) is a neurovisceral lysosomal storage disorder, characterized biochemically by a lipid trafficking defect resulting in intracellular accumulation of unesterified cholesterol and other compounds. With incidence estimates of 1∶120,000, it is a rare condition exhibiting an autosomal-recessive mode of inheritance. NPC is caused by homozygous or compound heterozygous mutations of either *NPC1* (95% of cases) or *NPC2* [1], [2]. The diagnosis of NPC is established by a combination of genetic and biochemical testing, which involves *NPC1*/*2* gene sequencing and the demonstration of impaired intracellular cholesterol transport by filipin staining, respectively [3]. The disorder presents with an extensive phenotypic variability, ranging from fatal neonatal disease to chronic neurological deterioration in late adulthood. Besides the key clinical feature vertical supranuclear gaze palsy (VSGP), neurological symptoms encompass ataxia, early-onset cognitive decline, psychiatric disturbances, and movement disorders [1], [2]. The majority of late-onset forms are diagnosed within the second or third decade, yet there are an increasing number of reported cases manifesting as late as 50 years or older, often mimicking common neurologic or psychiatric illnesses such as parkinsonian disorders or dementias [1], [2], [4]–[9]. As a result of its broad phenotypic spectrum, NPC is thought to be significantly under-diagnosed, which is momentous given that an orally applied enzyme inhibitor has proven to be an effective treatment option for slowing neurologic disease progression [1], [2], [10]. Recently, a remarkable proportion of NPC cases were found in adult patients with the concurrence of degenerative ataxia and presenile dementia (17% of 24 patients) [11]. Furthermore, corroborating the existence of an unrecognized pool of NPC, a multicentre study identified three NPC patients in 250 adult individuals (1.2%) suffering from psychosis and/or early-onset cognitive decline combined with neurological symptoms suggestive of NPC by *NPC1*/*2* gene sequencing [12]. To date, the prevalence of misdiagnosed NPC in populations with more common age-related neurodegenerative diseases is unknown.

On the molecular side, accruing evidence suggests that the group of lysosomal storage disorders or lysosomal dysfunction in general is linked with age-related neurodegenerative diseases such as Parkinson's disease (PD), frontotemporal lobar degeneration (FTLD), and progressive supranuclear palsy (PSP) [13]–[17]. Rare mutations in the lysosomal disorder genes *GBA* (Gaucher disease) and *SMPD1* (Niemann-Pick types A and B disease) were shown to represent susceptibility factors for PD [18], [19], and the fundamental involvement of the lysosome in PD pathogenesis is supported by the observation that known PD genes such as *SNCA*, *LRRK2*, *parkin*, *PINK1*, and *ATP13A2* regulate lysosome-dependent pathways or lysosomal activity [20]. In FTLD, a critical role of impaired lysosomal function was pinpointed recently as *TMEM106B*, a gene discovered as a FTLD risk factor in genome-wide association studies, was found to influence lysosomal function and morphology [21]. Moreover, major genetic forms of FTLD such as *PRGN* and *CHMP2B* encode proteins affecting the integrity of lysosome-dependent cellular processes [22], [23]. Finally, for the atypical parkinsonian disorder PSP, a recent genome-wide association study highlighted susceptibility at *STX6*, a gene implicated in the endosomal-lysosomal trafficking system, thus linking the disease to the lysosome as well [24].

Herein, in view of the clinical overlap and with regard to lysosomal dysfunction as a shared pathomechanistic feature, we screened for rare *NPC1* and *NPC2* sequence variants in patients clinically diagnosed with PD, FTLD and PSP, and a cohort of population-based controls. We first aimed to assess whether carriers of rare variants in *NPC1* and *NPC2* are at higher risk for developing PD, FTLD, or PSP. Second, based on genetic testing, we investigated the possibility of misdiagnosed NPC cases in the respective populations. Our analyses did not reveal any association between *NPC1*/*2* gene mutations and PD, FTLD, or PSP. Also, we could not identify any unrecognized NPC patients in our disease cohorts.

## Materials and Methods

### Standard protocol approvals, registrations, and patient consents

The study was approved by the ethics review board at the Technische Universität München, Munich, Germany, and the ethics review board of the Hessische Landesärztekammer in Frankfurt, Germany. All subjects provided a written consent form to participate in the study, which included detailed information about the genetic mutational screening and an authorization to publish the screening results. Subjects have been properly instructed and have indicated that they consent to participate by signing the appropriate informed consent paperwork. All potential participants who declined to participate or otherwise did not participate were eligible for treatment (if applicable) and were not disadvantaged in any other way by not participating in the study.

### Participants

The study population was composed of 563 patients diagnosed with PD (32.9% female, 69.4±6.8 years), 133 patients with FTLD (41.4% female, 63.8±8.2 years), 94 patients with PSP (42.6% female, 69.7±6.8 years), and 846 general population controls (47.8% female, 75.9±6.6 years). All patients were enrolled in one of three German Medical Centers specializing in neurodegenerative disorders (Department of Neurology and Department of Psychiatry, Klinikum rechts der Isar, Technische Universität München, Munich, Germany; Paracelsus-Elena-Klinik, Kassel, Germany; Department of Neurology, Klinikum der Universität München, Ludwig-Maximilians-Universität München, Munich, Germany). The clinical diagnoses were established according to the consensus criteria for PD [25], FTLD [26], and PSP [27]. General population controls belong to the KORA-AGE cohort, a subset of the original KORA survey enriched for older individuals [28]. Individuals with known dopaminergic medication or signs of neurodegenerative disease were excluded from the control sample. All participants of the study were Caucasian and originated from the same geographic region.

### Variant detection

Patients' blood samples were drawn and DNA was extracted from peripheral blood lymphocytes using standard protocols. PCR primers for the 25 exons and flanking intron regions of *NPC1* (RefSeq NM_000271) and the five exons and flanking intron regions of *NPC2* (RefSeq NM_006432) were designed with the ExonPrimer software (http://ihg.gsf.de). Primer sequences and PCR conditions are summarized in Tables S1 and S2. Variant screening was performed using Idaho®'s LightScanner™ high-resolution melting (HRM) curve analysis according to standard protocols (Idaho Technology Inc., Salt Lake City, UT) [29]. Samples with altered melting patterns were Sanger sequenced. In addition, Sanger sequencing of the entire *NPC1* or *NPC2* coding regions and flanking intron regions ensued when a known disease-associated mutation was identified, respectively. In our analyses, we focused on sequence variants with a minor allele frequency (MAF)<1% because NPC is known to be a rare condition caused by mutations with a very low frequency; synonymous substitutions were omitted since they are unlikely to be pathogenic.

### Statistical analysis

Differences in variant frequencies between cases and controls were analyzed using Fisher's exact test and statistical significance levels were set at *p*<0.05.

### In silico analysis of variants

PolyPhen2 [30], SIFT [31], and Mutation Taster [32] were used to evaluate the functional effect of single amino acid substitutions.

### Biochemical and laboratory investigations

The filipin test was performed on fibroblasts cultured from patient skin biopsies as previously described [33]. The slides were examined on a Nikon Eclipse 80i epifluorescence microscope using an UV-1A filter (excitation 365/10, DM400, BA 400) with narrow pass. Tests to aid the diagnosis of NPC comprise measurement of plasma chitotriosidase activity as well as assessment of certain cholesterol oxidation products in plasma (oxysterols) [1], [2]. Chitotriosidase activity was assayed using 4-methylumbelliferyl-β-D-N,N′,N″-triacetylchitotriose as a substrate [34]. Plasma levels of the oxysterol cholestane-3β,5α,6β-triol were quantified by gas chromatography-mass spectrometry as previously specified [35], [36].

## Results

In the present study, we identified rare sequence variants in *NPC1* and *NPC2* that had been previously found in patients with NPC and considered causative for the condition, henceforth referred to as “disease-associated”, as well as rare sequence variants of unknown significance. Table 1 details all known disease-associated variants in *NPC1* and *NPC2* observed in patients with PD, FTLD, PSP, and controls. The screening revealed four different disease-associated *NPC1* missense variants (p.Asn222Ser, p.Arg518Trp, p.Ser1004Leu, p.Pro1007Ala) in five independent individuals with PD and one possibly disease-associated *NPC2* missense variant (p.Val30Met) in an additional subject with PD, all in the heterozygous status, giving an overall variant frequency of 1.1% among PD cases. In contrast, no disease-associated variants were seen in the groups of FTLD and PSP patients. In the control cohort, seven heterozygous carriers (0.8%) of six different disease-associated *NPC1* variants were detected, including one nonsense, one small insertion and four missense mutations (p.Asn222Ser, p.Arg348X, p.F779fsX9, p.Ser1004Leu, p.Asn1156Ser, p.Arg1186His). All rare *NPC1*/*2* sequence variants of unknown significance, as detected in addition to known disease-associated mutations, are listed in Table 2. These alterations comprised a total of 16 different missense and five different tentative splicing variants, eleven of them novel (*NPC1*: p.Tyr157Cys, p.Thr477Met, p.His497Tyr, p.Ala521Pro, p.Asp611Gly, p.Pro974Leu, p.Val1158Met, c.1655-1G>A, c.2131-1G>C, c.3042-5C>T; *NPC2*: p.Pro46His); two of the variants (p.Asp611Gly, p.Val1158Met) were found in a single individual diagnosed with PD as described below. There were no significant differences in variant frequencies between patients with PD, FTLD, PSP and controls, neither for disease-associated *NPC1/2* variants alone nor for all rare variation found in the *NPC1* and *NPC2* genes (all *p*>0.05, Table 3).

**Table 1:** Disease-associated *NPC1* and *NPC2* variants detected in individuals with PD, FTLD, PSP, and KORA-AGE controls.

Gene	Exon	Variation nucleotide	Variation amino acid	Mutation type	dbSNP137	Freq PD (n = 563)	Freq FTLD (n = 133)	Freq PSP (n = 94)	Freq controls (n = 846)	Freq NHLBI-ESP (EA)	NPC disease-association reported in
NPC1	6	c.665A>G	p.Asn222Ser	missense	rs55680026	1	0	0	1	C = 52/T = 8548	[51]
NPC1	8	c.1042C>T	p.Arg348X	nonsense	not found	0	0	0	1	not found	[52]
NPC1	9	c.1552C>T	p.Arg518Trp	missense	not found	1	0	0	0	A = 2/G = 8598	[53]
NPC1	15	c.2336_2337insT	p.F779fsX9	frameshift	not found	0	0	0	1	not found	[54]
NPC1	20	c.3011C>T	p.Ser1004Leu	missense	rs150334966	2	0	0	2	A = 7/G = 8593	[54]
NPC1	20	c.3019C>G	p.Pro1007Ala	missense	rs80358257	1	0	0	0	C = 2/G = 8598	[55]
NPC1	22	c.3467A>G	p.Asn1156Ser	missense	rs28942105	0	0	0	1	not found	[56]
NPC1	23	c.3557G>A	p.Arg1186His	missense	rs200444084	0	0	0	1	T = 1/C = 8599	[56]
NPC2	2	c.88G>A	p.Val30Met	missense	rs151220873	1	0	0	0	T = 25/C = 8575	[57], [58]

Frequencies as found in the 4300 European American exomes of the NHLBI exome sequencing project (NHLBI-ESP, http://evs.gs.washington.edu/EVS/) are given for all identified variants. PD = Parkinson's disease; FTLD = frontotemporal lobar degeneration; PSP = progressive supranuclear palsy; Freq = frequency; EA = European American.

**Table 2:** Rare *NPC1/2* sequence variants of unknown significance detected in individuals with PD, FTLD, PSP, and KORA-AGE controls.

Gene	Exon/Intron	Genomic position (hg19)	Variation nucleotide	Variation amino acid	Mutation type	dbSNP137	Freq PD (n = 563)	Freq FTLD (n = 133)	Freq PSP (n = 94)	Freq controls (n = 846)	Freq NHLBI-ESP (EA)	PolyPhen2	SIFT	Mutation Taster
NPC1	2	chr18:21153416	c.180G>T	p.Gln60His	missense	rs145666943	0	0	1	2	A = 4/C = 8596	possibly damaging	N/A	disease causing
NPC1	5	chr18:21141485	c.470A>G	p.Tyr157Cys	missense		0	0	0	1	not found	probably damaging	damaging	disease causing
NPC1	5	chr18:21141414	c.541G>A	p.Ala181Thr	missense	rs199963560	0	0	0	1	not found	possibly damaging	damaging	disease causing
NPC1	9	chr18:21134845	c.1430C>T	p.Thr477Met	missense		0	0	0	1	not found	benign	tolerated	polymorphism
NPC1	9	chr18:21134795	c.1480G>A	p.Val494Met	missense	rs199812609	1	0	0	0	T = 1/C = 8599	benign	tolerated	disease causing
NPC1	9	chr18:21134786	c.1489C>T	p.His497Tyr	missense		0	0	0	1	not found	benign	tolerated	disease causing
NPC1	10	chr18:21131684	c.1561G>C	p.Ala521Pro	missense		0	0	0	1	not found	benign	tolerated	disease causing
NPC1	IVS11	chr18:21128073	c.1655-1G>A		(near-)splice		0	0	0	1	not found	N/A	N/A	disease causing
NPC1	11	chr18:21128055	c.1672G>T	p.Ala558Ser	missense	rs201156397	0	0	1	0	not found	probably damaging	damaging	disease causing
NPC1	12	chr18:21125039	c.1832A>G*	p.Asp611Gly	missense		1	0	0	0	not found	probably damaging	damaging	disease causing
NPC1	IVS14	chr18:21123534	c.2131-1G>C		(near-)splice		0	0	0	1	not found	N/A	N/A	disease causing
NPC1	16	chr18:21121118	c.2428G>T	p.Val810Phe	missense	rs145362908	1	1	0	1	A = 5/C = 8595	benign	tolerated	disease causing
NPC1	IVS20	chr18:21118640	c.2912-5G>A		(near-)splice		1	0	0	0	T = 1/C = 8599	N/A	N/A	polymorphism
NPC1	20	chr18:21118626	c.2921C>T	p.Pro974Leu	missense		0	0	0	1	not found	benign	tolerated	disease causing
NPC1	IVS21	chr18:21116845	c.3042-5C>T		(near-)splice		1	0	0	0	not found	N/A	N/A	polymorphism
NPC1	21	chr18:21116665	c.3217G>A	p.Gly1073Ser	missense	rs141440861	4	0	1	4	T = 16/C = 8584	benign	tolerated	disease causing
NPC1	22	chr18:21115438	c.3472G>A*	p.Val1158Met	missense		1	0	0	0	not found	probably damaging	damaging	disease causing
NPC2	2	chr14:74953085	c.137C>A	p.Pro46His	missense		0	1	0	0	not found	probably damaging	tolerated	disease causing
NPC2	3	chr14:74951269	c.212A>G	p.Lys71Arg	missense	rs142075589	0	1	0	1	C = 2/T = 8598	possibly damaging	tolerated	disease causing
NPC2	3	chr14:74951189	c.292A>C	p.Asn98His	missense	rs142858704	1	0	0	2	G = 10/T = 8590	benign	tolerated	polymorphism
NPC2	IVS4	chr14:74947404	c.441+1G>A		(near-)splice	rs140130028	2	1	0	1	T = 76/C = 8524	N/A	N/A	disease causing

Frequencies as found in the 4300 European American exomes of the NHLBI exome sequencing project (NHLBI-ESP, http://evs.gs.washington.edu/EVS/) are given for all identified variants. Additionally, in silico predictions of the damaging potential of all variants assessed by PolyPhen2, SIFT, and Mutation Taster are noted.

identified in the same individual.

PD = Parkinson's disease; FTLD = frontotemporal lobar degeneration; PSP = progressive supranuclear palsy; Freq = frequency; EA = European American; N/A = not applicable.

**Table 3:** *NPC1/2* variant frequencies by group^a^.

	PD	FTLD	PSP	Controls
	No.(%)	No.(%)	No.(%)	No.(%)
	(n = 563)	(n = 133)	(n = 94)	(n = 846)
Disease-associated variants^b^	6 (1.1%)	0	0	7 (0.8%)
Fisher's exact test	*p* = 0.78	*p* = 0.6	*p* = 1.0	reference
all rare variants^c^	18 (3.2%)	4 (3.0%)	3 (3.2%)	26 (3.1%)
Fisher's exact test	*p* = 0.88	*p* = 1.0	*p* = 1.0	reference

PD = Parkinson's disease; FTLD = frontotemporal lobar degeneration;

PSP = progressive supranuclear palsy.

^a^ Absolute number of variant carriers, percentage of carriers within the group, *p* values.

^b^ Variants previously described as disease-causing in a NPC patient.

^c^ All rare (MAF<1%) variants detected in *NPC1* and *NPC2* (synonymous changes omitted).

The clinical characteristics of the six PD patients that were heterozygous for disease-associated *NPC1*/*2* variants are shown in Table 4. The age of disease onset ranged from 55 to 76 years, with an average onset at 65.8 years. All patients presented typical parkinsonian features responsive to dopaminergic agents, four patients exhibited a marked cognitive decline with disease progression, and two patients had a positive family history for PD. Over the course of disease, two patients were noted to have impaired eye movements, patient number 2 both vertical and horizontal and patient number 4 vertical. Patient number 4 further developed psychiatric symptoms at an early disease stage (Table 4). Sanger sequencing of the entire *NPC1* or *NPC2* coding regions and flanking intron regions detected no additional rare variants in these six individuals, respectively.

**Table 4:** Clinical characteristics of PD patients carrying disease-associated *NPC1/2* variants.

Patient No./Sex	Genotype	Age of onset, y	Age at sampling, y	IS	B	R	RT	PI	D	L-Dopa/DA	Family history	Additional information
1/M	p.Asn222Ser/wt (NPC1)	55	65	B	+	−	+	+	−	+	positive	none
2/M	p.Arg518Trp/wt (NPC1)	60	80	B	+	+	+	+	+	+	negative	vertical and horizontal gaze impaired
3/M	p.Ser1004Leu/wt (NPC1)	76	87	RT	+	+	+	−	−	+	negative	none
4/F	p.Ser1004Leu/wt (NPC1)	75	79	B	+	+	−	+	+	+	negative	vertical gaze impaired, hallucinations early in the disease course
5/M	p.Pro1007Ala/wt (NPC1)	61	72	B	+	+	+	−	+	+	positive	none
6/M	p.Val30Met/wt (NPC2)	68	71	B	+	+	−	+	+	+	negative	none

PD = Parkinson's disease; Sex: M = male, F = female; wt = wild type; IS = initial symptom; B = bradykinesia; R = rigor; RT = resting tremor; PI = postural instability; D = dementia; DA = dopamine agonist.

Overall, the screening disclosed no rare *NPC1*/*2* variants either in homozygosity or in compound heterozygosity, hence no NPC cases were recognized by our genetic analyses. One patient diagnosed with PD was found to carry two novel heterozygous missense variants in *NPC1* (p.Asp611Gly, p.Val1158Met), both with consistent pathogenic prediction by three prediction programs used (SIFT, PolyPhen2, Mutation Taster; Table 2). Segregation analysis demonstrated that the variants were not inherited independently but resided on the same chromosome. Thus, the patient was not compound heterozygous for the variants and did not meet the NPC diagnostic criteria [3]. Filipin test performed in cultured skin fibroblasts of this subject showed a pattern resembling the “variant” biochemical phenotype of NPC (Figure S1) [33]. Chitotriosidase activity and plasma oxysterol levels were in the normal range. Clinically, the 60-year-old man suffered from PD since the age of 55 years with markedly left-sided bradykinesia, rigidity, and rest tremor, and an excellent response to dopaminergic medication. There were no atypical signs for PD. Family history was positive for neurodegenerative disorders with his mother having been diagnosed with Alzheimer's disease, but negative for any movement disorders.

## Discussion

We investigated the possible role of rare sequence variants in the *NPC1* and *NPC2* genes, mutations in which are causative for the lysosomal storage disorder NPC, in three age-related neurodegenerative diseases (PD, FTLD, PSP). Dysfunction of the lysosomal degrading system has been implied in a variety of neurodegenerative processes and lysosomal storage disorders in particular have been strongly linked to parkinsonism [13]–[17], [20]. Mutations in the *GBA* gene, which encodes the lysosomal enzyme deficient in Gaucher disease, are one of the commonest risk factors for PD, which was primarily shown in Ashkenazi Jewish individuals and subsequently in a number of other populations worldwide [37]–[41]. More recently, a founder mutation in *SMPD1*, the gene for Niemann-Pick types A and B disease (acid sphingomyelinase deficiencies), was recognized as a novel susceptibility factor for PD in the Ashkenazi Jewish population [19]. The same study failed to prove association between PD and founder mutations in the lysosomal enzyme genes *HEXA* (Tay-Sachs disease) and *MCOLN1* (mucolipidosis type IV) [19]. Now, our analyses generated evidence that mutations in the lysosomal storage disorder genes *NPC1* and *NPC2* are not associated with PD in a homogeneous sample of European descent. The proportion of PD patients positive for disease-associated *NPC1*/*2* variants (1.1%) was relatively low when compared to *GBA* mutation frequencies reported in non-Jewish PD cohorts (4–7%) [18], [42]. Moreover, rare variants in *NPC1*/*2* appear not to be associated with FTLD and PSP in the German population. Notably, there are limitations to the present study. First, the study was powered at 80% to detect a significant association of rare *NPC1*/*2* variants with PD when modeling odds ratios ≥2.08 (significance level of 0.05, cumulative MAF of rare variants in the present study ∼1.6%). The sample size was not large enough to judge modest or small effects of rare *NPC1*/*2* variants on PD risk. Taking this further, for an association with FTLD or PSP odds ratios should have been ≥3.86 (power of 80%), considering that these patient cohorts were relatively small. Second, our control sample was composed of individuals from the general population without signs of neurodegenerative diseases or taking dopaminergic drugs. Nonetheless, there might be potential risks for PD, FTLD, and PSP later in life and these could have confounded our observations. The carrier frequency for disease-associated *NPC1* variants among control subjects (0.8%) is in line with the predicted frequency of 0.6% given a disease incidence of 1∶120,000. Third, as we used HRM for variant detection and did not perform Sanger sequencing of the entire *NPC1*/*2* coding region, the frequencies of *NPC1*/*2* variants could be underestimated across all samples. However, this effect was likely to be small since previous investigations applying HRM yielded a diagnostic sensitivity of 100% for heterozygous variants and 93% for homozygous variants [29], [43]. Ultimately, different results may be obtained by using more specific inclusion criteria for patients like an early disease onset or a positive family history, or by conducting the study in geographically and ethnically different populations.

Albeit the lack of a genetic association in this study, it cannot be fully excluded that heterozygous pathogenic variants in *NPC1* and *NPC2* represent a component of risk for age-related neurodegenerative disorders or might play a role in certain subsets of such patients. In two of six PD patients (33%) heterozygous for disease-associated *NPC1/2* variants impaired vertical gaze was found on clinical examination, an atypical sign for PD and the key feature of NPC, and one of these patients developed concomitant psychiatric symptoms early in the disease course. Findings from animal models highlight that heterozygous *NPC1* mutations affect neuronal function and neurodegenerative disease status, particularly in the context of aging [44], [45]. Further, several studies suggest the possibility of symptomatic heterozygotes in human NPC: Josephs *et al.* proposed one mutant *NPC1* allele as the cause of parkinsonian tremor in a 75-year-old patient [46]. Harzer *et al.* report a *NPC1* heterozygote manifesting systemic signs of NPC during childhood [47]. And, a very recent manuscript described three independent adult relatives of NPC patients who were heterozygous *NPC1* mutation carriers and exhibited a parkinsonism syndrome [48].

NPC displays an extreme clinical heterogeneity, with a large number of possible differential diagnoses. The most common presentation in adult-onset cases is a psychiatric illness combined with cognitive decline and motor signs (parkinsonism in 10%), but mild clinical pictures with predominant motor dysfunction are also observed [5]. VSGP is a characteristic sign of NPC, but also evident in other neurological disorders. In the present study, we could not unveil any misdiagnosed NPC in 563 patients with PD, 133 patients with FTLD, and 94 patients with PSP by means of a mutational screen. This negative result notwithstanding, it seems important to note that NPC patients might be identified in adult neurologic disease cohorts, for example when testing larger numbers of patients or including individuals exhibiting more exceptional clinical presentations, as recently demonstrated [11], [12]. Besides, it is possible that NPC diagnoses could have been missed because sensitivity of HRM is not 100%, and there was no exploration of large deletions or deep intronic mutations, which were shown to be rarely responsible for NPC [49], [50] [Latour and Vanier, unpublished data]. Our study detected an individual with PD who carried two novel *NPC1* missense variants (p.Asp611Gly, p.Val1158Met) but was found not to be a compound heterozygote on segregation analysis. Notably, this case emphasizes the crucial need to check for independent allele segregation when establishing the diagnosis of NPC by gene sequencing. Biochemical characterization of the two novel variants by filipin staining revealed that at least one or the combination of the two variants is functionally relevant to the NPC1 protein since mild abnormalities resembling the “variant” biochemical phenotype were observed in patient skin fibroblasts. This pattern is seen in a subset of patients with NPC but is also well documented in heterozygote carriers of NPC [1], [33]. An effect on plasma oxysterol levels has been described for heterozygous *NPC1* mutations [35], but was not seen in this case. Moreover, confirming the polymorphic nature of the NPC loci, our study disclosed eight additional novel variants in *NPC1* and one novel variant in *NPC2*, yet their functional significance with regard to the NPC1 and 2 proteins remains unknown.

In conclusion, our study indicates that rare variants in the *NPC1* and *NPC2* genes are not associated with PD, FTLD, and PSP in our populations and that, moreover, misdiagnosed NPC seems not to be frequent in these entities. Further *NPC* mutational screenings in larger and ethnically diverse cohorts of patients with PD and other neurodegenerative conditions should be undertaken to conclusively define the contribution of these lysosomal genes to the development of age-related neurodegeneration.

## Supporting Information

Figure S1 Filipin test from a PD patient carrying in cis the *NPC1* variants p.Asp611Gly and p.Val1158Met. Fibroblasts cultured from skin biopsies of a healthy control subject (negative control, **A**), a classical NPC patient (positive control, **B**), and the PD patient (**C**), after staining of unesterified cholesterol by filipin. The fibroblasts were maintained three days in a culture medium supplemented with 10% lipoprotein-deficient calf serum to maximize LDL-receptors expression. The cholesterol-starved fibroblasts were then challenged with human purified LDLs (50 µg/ml medium) for 24 h, and finally fixed with formalin and stained [33]. Cells were examined by epifluorescence microscopy (Nikon Eclipse 80i, UV-1A filter, ×20 Planfluor objective, DXM1200-C/NIS Elements imaging system). In C, the PD patient presents 30–50% of weakly positive cells. Original magnification ×200.
(TIF)

Table S1 Primers used for HRM and Sanger sequencing.
(DOC)

Table S2 Touchdown PCR protocol.
(DOC)
