# Supplementary figures and images for: Niemann-Pick C Disease Gene Mutations and Age-Related Neurodegenerative Disorders
Source: PLoS One. 2013 Dec 30;8(12):e82879. doi: 10.1371/journal.pone.0082879 (PMC3875432; doi:10.1371/journal.pone.0082879)

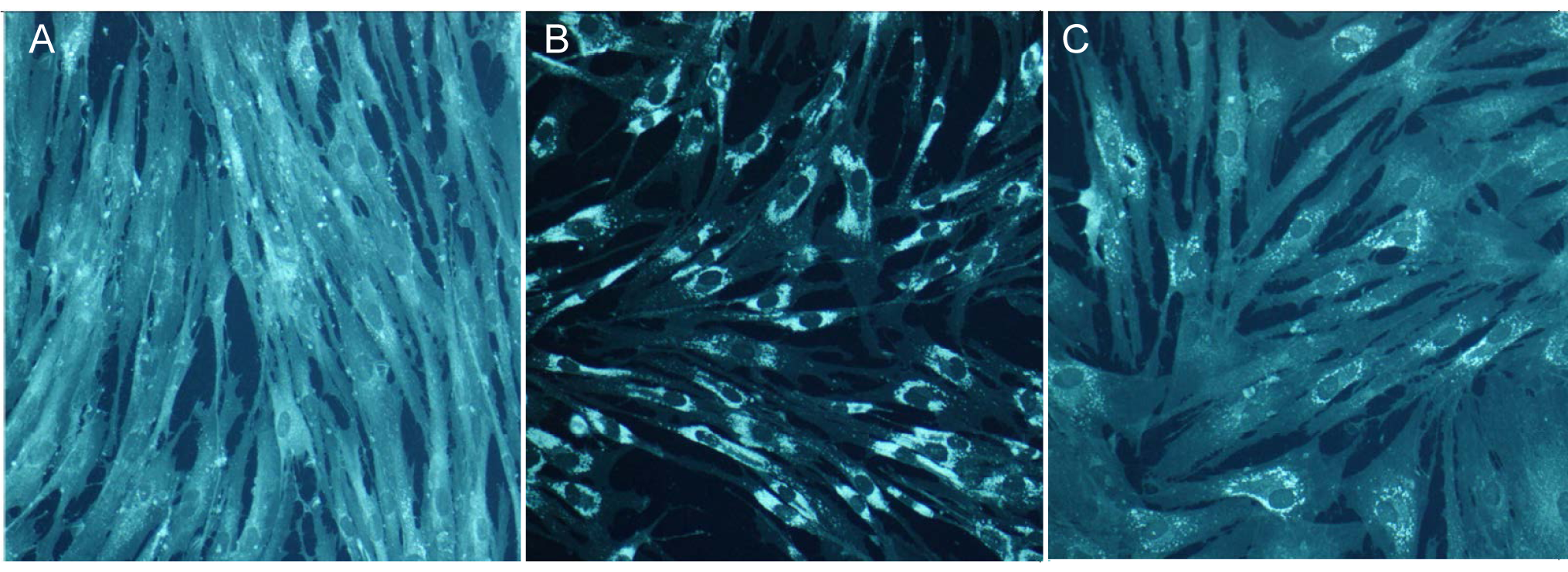

Supplement: Figure S1 — Filipin test from a PD patient carrying in cis the NPC1 variants p.Asp611Gly and p.Val1158Met. Fibroblasts cultured from skin biopsies of a healthy control subject (negative control, A), a classical NPC patient (positive control, B), and the PD patient (C), after staining of unesterified cholesterol by filipin. The fibroblasts were maintained three days in a culture medium supplemented with 10% lipoprotein-deficient calf serum to maximize LDL-receptors expression. The cholesterol-starved fibroblasts were then challenged with human purified LDLs (50 µg/ml medium) for 24 h, and finally fixed with formalin and stained [33]. Cells were examined by epifluorescence microscopy (Nikon Eclipse 80i, UV-1A filter, ×20 Planfluor objective, DXM1200-C/NIS Elements imaging system). In C, the PD patient presents 30–50% of weakly positive cells. Original magnification ×200. (TIF) [file pone.0082879.s001.tif]
