# Supplementary material for: Niemann-Pick C Disease Gene Mutations and Age-Related Neurodegenerative Disorders
Source: PLoS One. 2013 Dec 30;8(12):e82879. doi: 10.1371/journal.pone.0082879 (PMC3875432; doi:10.1371/journal.pone.0082879)
Supplement: Table S1 — Primers used for HRM and Sanger sequencing. (DOC) [file pone.0082879.s002.doc]

| Gene | Exon | Forward Primer | Reverse Primer | Annealing Temp. |
| --- | --- | --- | --- | --- |
| ***NPC1*** | ex1 | AACAGCCCGGGGAAGTAG | CTCCATCGCCAGACCAAC | 62° |
|  | ex2 | TGAGTGGGCACTTCTTGTTG | CACCTCCACCCTGCAATAAC | touchdown |
|  | ex3 | TGAGGAATGTTGACCTTACTCTAAC | GAAAGCTGAGCATTACCAGTTC | touchdown |
|  | ex4 | TTTAAAATCGTTCTTGCTGGC | CAATTTGCTCTGCTGTCCTG | touchdown |
|  | ex5 | TCTTGCCTCGTGAATTACAGC | ACTGTGCCCAGCCAGTTC | touchdown |
|  | ex6 | TTCAGTGGGCTTTTCTTTGAG | CAAATGAAAGCTCAAAGTGCC | touchdown |
|  | ex7 | TGGGATTACAGGAATGTCCC | GCAACCCCACTGAGGAAAC | touchdown |
|  | ex8 | ACTTTCAGGAACGGCTTGG | CATGTAAAAGCCAGCAAACC | touchdown |
|  | ex9 | TGACCCTCAGGGCAATG | TTTGCTCACCTCTGGGTTATG | touchdown |
|  | ex10 | AGGGCCCATGTTGTCCTTAG | GGTAAGAAATTAACAAAACTGCCC | touchdown |
|  | ex11 | CCCCTGGTATGTGTCTAATTTTC | AAGTGTCTAGCTTCCCACAATG | touchdown |
|  | ex12 | AAAACGTGGCCTTTGTATCG | GAAAATAGATGTAGGCAACAGAAAC | touchdown |
|  | ex13 | AAGTGGGACAGACAACCCTG | CAGGAGCCATTCACAGTCC | touchdown |
|  | ex14 | ACACAAGGCAGCAAGAAATG | AGCTCCTTCTTTCTCCAGGC | touchdown |
|  | ex15-16 | CATGAACATAAGACCTGCAGAGAG | TCTTAGAAGGCATGTGATAATCTG | touchdown |
|  | ex17 | TGCTTAAGATTTTATTTCTGGTCG | AGCAGGCACTTGCTTGAAAC | touchdown |
|  | ex18 | GATCCTCGCCTTGCTTAGTTAC | GACTGCCTGGCTGAGAGC | 63° |
|  | ex19 | TGAAACTAAAGACTTCCTCCCTG | CAAATAGGTATAAACTGAGGCACG | touchdown |
|  | ex20 | AAGAAAGTAATGCCCCTCACTG | CCATGCAACTGTCTTAGCCC | touchdown |
|  | ex21 | TTTGCTTAGCCTCAAGTGCTC | ACCCAGTGTAGGCCCTTTG | touchdown |
|  | ex22 | GTGACAGGATGAACACGCAG | TCTAAGACAGCCAATTCCCC | touchdown |
|  | ex23 | TGCCTAGGGTCTTCAACAGG | GAGGAGGATTACTTTGTGGTGC | touchdown |
|  | ex24 | CTTGAACCTGGGAGAAATCC | TCAGGATAGAATTCCCTTTCAG | touchdown |
|  | ex25 | TATGCCCAGCCAACTAGCTC | AGTGGTAAACCGACCGACC | 60° |
| ***NPC2*** | ex1 | ACAGGTTTGTCTTGTGACCG | AGCCCCAGGGGTCTCAG | 61° |
|  | ex2 | GCCTAGCTGGCTTATTTCTCC | TCCATTCCCATGCTTATTCC | touchdown |
|  | ex3 | GACCCTAGGAATGCTGTTGC | CCCATCTCTGCTTCTTGCC | touchdown |
|  | ex4 | CCAATTATGGATAGAAGTCAGGTC | TTTCTCCTCCACTTTCTTCCC | 60° |
|  | ex5 | CTTGCCCTAGGGTTATTGCC | GGTGCTGTCAAGAGTCTCAGC | 58° |

**Table S1** Primers used for HRM and Sanger sequencing
