# Supplementary material for: Niemann-Pick C Disease Gene Mutations and Age-Related Neurodegenerative Disorders
Source: PLoS One. 2013 Dec 30;8(12):e82879. doi: 10.1371/journal.pone.0082879 (PMC3875432; doi:10.1371/journal.pone.0082879)
Supplement: Table S2 — Touchdown PCR protocol. (DOC) [file pone.0082879.s003.doc]

**Table S2** Touchdown PCR protocol

| 95°C | 15 min | 1 cycle |
| --- | --- | --- |
| 95°C | 30 sec |  |
| 65°C to 53°C | 30 sec | 24 cycles |
| 72°C | 60 sec |  |
| 95°C | 30 sec |  |
| 54°C | 30 sec | 24 cycles |
| 72°C | 60 sec |  |
| 72°C | 10 min | 1 cycle |
| 25°C | 30 sec | 1 cycle |
